# Collage technique may provide new perspectives for Alzheimer patients by exploring messages from their inner world

**DOI:** 10.1590/S1980-57642009DN30400006

**Published:** 2009

**Authors:** Mitsue Meguro, Junichi Ishizaki, Kenichi Meguro

**Affiliations:** 1 RN, MSc, Department of Geriatric Behavioral Neurology, Tohoku University Graduate School of Medicine.; 2 PhD, Department of Geriatric Behavioral Neurology, Tohoku University Graduate School of Medicine.; 3 MD, PhD, Department of Geriatric Behavioral Neurology, Tohoku University Graduate School of Medicine.

**Keywords:** Alzheimer’s disease, collage technique, spiritual care

## Abstract

Although the collage art technique has been introduced as a psychotherapeutic method, it has not been fully applied in dementia.

**Objectives:**

To analyze characteristics of the collage articles produced by patients with Alzheimer’s disease (AD).

**Methods:**

Twenty AD patients were asked to select and place several clippings as they wished. The MMSE was used for cognitive assessments.

**Results:**

Simplification and poor organization in their articles were found. The themes of one patient were found to change according to behavior. We discussed the images of the articles, especially spiritual images in the early stage and family images in the later stage.

**Conclusions:**

We concluded that the collage technique could provide new perspectives for dementia patients by exploring messages from their inner world.

## Abstract (pt)

Embora a técnica de colagem tenha sido introduzida como um método psicoterapêutico, ela não foi totalmente aplicada em demência.

**Objetivos:**

Analisar as características de trabalhos de colagens feitos por pacientes com doença de Alzheimer (DA).

**Métodos:**

A 20 pacientes foi solicitado que selecionassem e colocassem peças que apreciassem. O MEEM foi utilizado para avaliação cognitiva.

**Resultados:**

Simplificação e organização pobres foram encontradas. Nós demonstramos um paciente cujos temas foram mudando em relação ao comportamento. Nós discutimos as imagens dos artigos, especialmente imagens espirituais no estágio precoce e imagens familiares no estágio tardio.

**Conclusões:**

Nós consideramos que a técnica de colagem poderia dar novas perspectivas na demência, explorando a mensagem do mundo interior.

Collage is well known as a unique technique of modern art. In academic history, it was first introduced as a method of assessing the psychodynamics of psychiatric patients,^1^ and has become popular in art therapy.^2^ It is sometimes used in psychosocial interventions for patients with Alzheimer’s disease (AD) as a part of creative and recreational activities.^3^ In their book on psychological support for elders with dementia, Holden et al.^4^ noted “these can be made in the group; members search through magazines looking for pictures that illustrate the particular theme, cut these out and stick them on a large piece of paper”(pg. 159-160).

Although collage activities have become popular among therapists working on dementia in Western countries, the expressions of resulting articles have not been fully analyzed. In Japan, the collage technique was introduced as a psychotherapeutic “collage therapy” in the late 1980s. It has since been applied in various illnesses such as neurosis, depression, schizophrenia, etc.^5^ In Japanese reports, therapists have generally interpreted the expressions of individuals in terms of the framework of projective or symbolic theory. One of the present authors (J.I.) has applied it in an assessment and therapeutic method for dementia and reported the form and content analyses.^6^ We also recorded the themes in the collage articles shown by typical AD cases.^7^

In this article, the expression of collage articles produced by AD patients was analyzed. First, we reported specific difficulties in making collage as well as the characteristics of deficits in the features of form of expression with reference to patients’ neuropsychological impairments. Second, we report the expressed content by illustrating with several typical articles and showing the changing of the theme in the serial articles. We also discuss the images in the context of the disease process.

## Patients and methods

Twenty patients (3 men and 17 women, mean age of 78.5 years) were diagnosed as possible or probable AD based on the NINCDS-ADRDA criteria.^8^ A sheet of drawing paper from B5 to A3 size was used. The patients were asked to select and place several clippings in the manner they wished. The pieces had been cut out from old magazines by patients when their symptoms were relatively mild. The pieces were cut out in advance and put in a box by therapists for patients with severe dementia. The Mini-Mental State Examination (MMSE)^9^ was used for cognitive assessments. The mean MMSE score was 15.2.

## Results

### Characteristics of form features

The AD patients often did not start the collage procedure spontaneously. When asked which pictures they liked, they were able to point out them. However, especially early in the process of making collage, most of them needed some support from therapists. During the initial sessions, they learned about making collage. This phenomenon may be due to patients’ difficulty in responding to an unfamiliar situation, as well as their decline in executive function, which is needed for planning and manipulation of sequential procedures.

**Figure 1:**
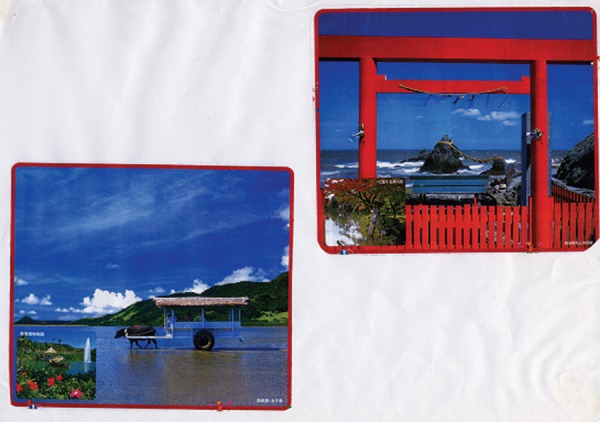
An example of simplification.

Many cases in this study showed a tendency to simplify, i.e., they cut several pieces into squares and placed them parallel to one another on the sheet of paper as shown in Figure 1.

### Characteristics of content features

**Figure 2:**
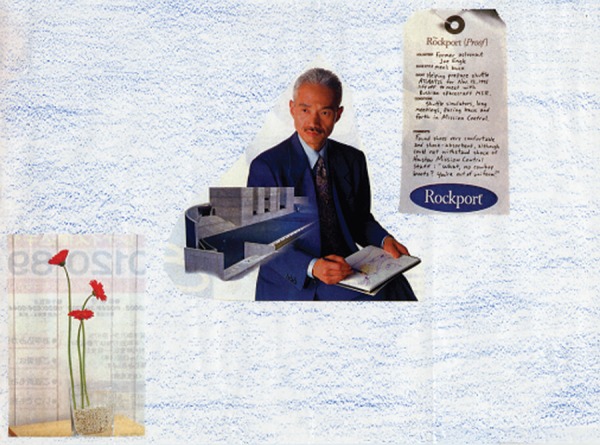
Theme: school and teacher.

Figure 2 illustrates an article produced by a 72-year-old woman. She made a collage article for the first time. Her MMSE score was 15. Her condition gradually worsened, and her latest MMSE score fell to 10 (2 years later).

In reference to the article, she explained that the person in the center was a scholar. Thus, she stated that she needed to put an English document beside him. She felt a tense atmosphere, and so added a flower to create some feeling of relief. In the next collage article, she expressed the “school and teacher” theme. In these images of her early articles, it is evident from her inner world that she was specifically alluding to her career as a teacher and that she was always proud of her ability to speak English.

The patient produced several subsequent articles on the same theme the following year. After approximately 1 year, the theme changed from “school and teacher” to “mother and child.” A little girl was depicted toddling in the center of the image. A young lady was depicted sat holding a big tube-like cushion and smiling to the left side. The patient explained that she was a mother. A mother was nursing the baby in the right corner. She stated “I want to put a child in the center. There is a mother too. And the mother is nursing the baby!”

Interestingly, in parallel, we observed changes in her daily behavior together with the collage theme. Namely, she had a stern look and began complaining to the staff for the first time. She did, however, join in with group session in a good mood and assisted other patients. Her complaints to the staff dwindled. Her self-image conveyed in her speech also changed, i.e., from a very strict teacher to a kind lady who had never got cross with students. The collage images clearly showed her inner change.

In the serial articles, the first theme “school and teacher” seemed to directly reflect her life history. The later theme “parent and child” also appeared to draw on her life history. She had lived with two daughters in her early life and had become separated from them later. This can usually be interpreted as a result of the severe retrograde amnesia caused as the disease progresses. However, in contrast to a “teacher,” she had never spoken on the topic of “child.” This was expressed repeatedly only through collage articles, and thus she may not have been aware of the theme. We discussed this point from another angle.

**Figure 3:**
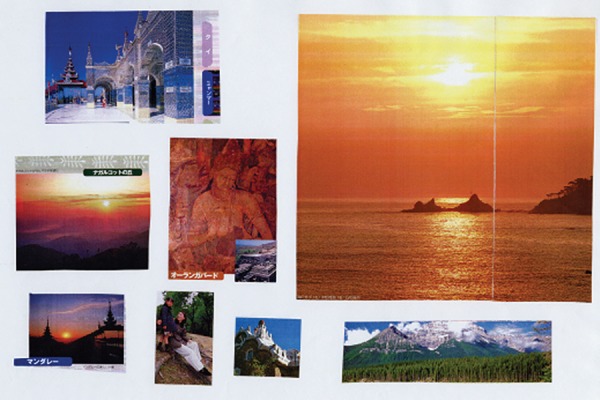
Spiritual image.

For patients in the early stage, we found a spiritual theme regarding preparations for death as a last life task. Figure 3 is an article by an 82-year-old woman. Her MMSE score was 23. The main items were the sunrise or sunset and an image of Buddha. These expressions were thought to be simply on a spiritual theme. Butler^10^ noted, “old age inaugurated the process of the life review, promoted by the realization of approaching dissolution and death” (pg. 534). These processes may be promoted by the awareness of the disease in the early stage in AD patients. Intriguingly, the spiritual theme was generally not spoken of in conversations with patients yet was shown in their collage articles.

## Discussion

### Characteristics of the form features

Simplification and poor organization were also reported in previous studies on drawing impairments in AD^11,12^ in a similar fashion to those observed in the form analysis of the collage expression in our patients. These deficits were related to executive dysfunction in the frontal lobe and visuospatial dysfunction in the parietal lobe.^6,7,11,12^

Although AD patients had some difficulties in making collage, collage is easier than drawing. The activity depends mainly on cognitive functions which remain relatively intact in AD patients such as semantic memory and procedural memory.^13^ In fact, through practice, many of our patients learned to make articles more proficiently and rapidly.

### Characteristics of the content features

Although the patients had severe amnesia, we frequently found consistent expression of the same theme in the serial collage articles. Several hours later, patients were unable to remember clearly that they had produced the articles. Therefore, this makes it less conceivable that they had intended to produce series on the same particular theme. The themes might have reflected some special memory in their lives such as occupation.^14^

### Further comments

In the last decade, several authors have directed their attention to investigating more internal aspects of dementia. Sabat et al.^15^ reported detailed discourse of conversation with AD patients and emphasized that a sense of self was intact in the world, and even if in the severe stage of the disease, some patients were still aware of their deficits. Bender et al.^16^ noted that psychologists should understand the subjective world of dementia patients and that the application of various psychotherapeutic methods was necessary. Holden et al.^4^ described their psychological interventions as an integrated care approach for dementia, and Clare et al.^17^ discussed the possibility of cognitive rehabilitation in dementia. Moreover, Coleman et al.^18^ discussed the significance of the spiritual aspect in care. All these arguments can be interpreted as an attempt to construct a more holistic perspective for dementia. We believe the collage method, as a psychological technique, can contribute to this new perspective for dementia patients by exploring the message from the inner world of dementia patients.
